# Research evaluation support services in biomedical libraries

**DOI:** 10.5195/jmla.2018.205

**Published:** 2018-01-02

**Authors:** Karen Elizabeth Gutzman, Michael E. Bales, Christopher W. Belter, Thane Chambers, Liza Chan, Kristi L. Holmes, Ya-Ling Lu, Lisa A. Palmer, Rebecca C. Reznik-Zellen, Cathy C. Sarli, Amy M. Suiter, Terrie R. Wheeler

## Abstract

**Objective:**

The paper provides a review of current practices related to evaluation support services reported by seven biomedical and research libraries.

**Methods:**

A group of seven libraries from the United States and Canada described their experiences with establishing evaluation support services at their libraries. A questionnaire was distributed among the libraries to elicit information as to program development, service and staffing models, campus partnerships, training, products such as tools and reports, and resources used for evaluation support services. The libraries also reported interesting projects, lessons learned, and future plans.

**Results:**

The seven libraries profiled in this paper report a variety of service models in providing evaluation support services to meet the needs of campus stakeholders. The service models range from research center cores, partnerships with research groups, and library programs with staff dedicated to evaluation support services. A variety of products and services were described such as an automated tool to develop rank-based metrics, consultation on appropriate metrics to use for evaluation, customized publication and citation reports, resource guides, classes and training, and others. Implementing these services has allowed the libraries to expand their roles on campus and to contribute more directly to the research missions of their institutions.

**Conclusions:**

Libraries can leverage a variety of evaluation support services as an opportunity to successfully meet an array of challenges confronting the biomedical research community, including robust efforts to report and demonstrate tangible and meaningful outcomes of biomedical research and clinical care. These services represent a transformative direction that can be emulated by other biomedical and research libraries.

## INTRODUCTION

Over the past few decades, libraries supporting research-intensive universities, major health institutes, and medical schools have found themselves entering a new, dynamic environment. Specific developments in information access, organization, and services have made libraries key players in tracking the dissemination and impact of research, clinical care, and teaching in the biomedical domain.

The implementation and use of increasingly sophisticated literature databases, repositories, content management systems, and research networking platforms afford libraries access to a vast array of digital data. These data are both qualitative and quantitative and include bibliographic data, survey data, gray literature, altmetrics and social media data, and grant funding data. Given their expertise in discovering, capturing, describing, analyzing, curating, and visualizing data, librarians are well qualified to develop and promote innovative approaches to biomedical data management, analysis, and visualization.

The rise in evidence-based decision making and the increased demand for evaluation of research [1] has led many libraries to develop research evaluation support services. Libraries can serve as neutral but active participants in an evaluation setting by proposing reliable measures, providing appropriate data, and reinforcing responsible use of metrics [2]. In the broad evaluation landscape, libraries are involved in many types of projects, and generally, these projects focus on research output or impact evaluation. They assist universities in assessing the dissemination of their research and evaluating success in meeting the university’s core goals. Libraries help departments track their output or fairly evaluate their faculty in promotion and tenure decisions [3]. Libraries also provide guidance for researchers on better communicating the impact of their work in grant applications or creating successful dissemination plans for their research.

There have been significant advances in research assessment over the past two decades, beginning with the development of the “Payback Framework” [4], which examined the impact of health services research in the United Kingdom. Since then, funding bodies, universities, and even libraries have piloted and developed research assessment frameworks, such as the Becker Medical Library Model for Assessment of Research Impact [5] and the Canadian Academy of Health Sciences framework [6]. These frameworks are often applied to assess how research has benefited key groups, to steer research toward desired outcomes, to show effectiveness or ability to conduct research, to reward innovative research, and to increase accountability of researchers, funding bodies, and policy makers by being transparent about the research process [1].

Library-led research evaluation support services are increasingly common in European and Australian contexts where large-scale research evaluation exercises have necessitated a response. A recent study of 140 libraries in Ireland, New Zealand, Australia, and the United Kingdom showed that the majority offer bibliometric training or literacy as well as citation reports [7]. Many US and Canadian libraries have also implemented diverse models to support evaluation-based activities. A recent study of Association of Research Libraries member libraries found that seventy-six of the seventy-nine responding libraries reported that they provided services related to evaluation of research impact and that these services represented a growth area for their libraries [8]. Additionally, a review of the library websites of the sixty-two prestigious Association of American Universities members found that only one library did not provide users with information about research metrics and impact [9].

In this paper, the authors discuss the experiences of seven US and Canadian libraries in providing research evaluation support services to their customer groups. Each library completed a basic questionnaire so that we could capture and aggregate our shared knowledge, discuss experiences with establishing research evaluation support services, and examine future plans. We discuss the unique context of each library, the types of services that the libraries provide, and how the libraries utilize various marketing techniques. Furthermore, we list their collaboration partners and describe the combinations of resources and tools that each library uses to accomplish their work.

## BUILDING OF A NETWORK OF COLLEAGUES

Because research evaluation support is a more recently implemented service provided by libraries, many librarians may feel that they have only had brief exposure to some of the most useful tools or the most basic training in important concepts such as bibliometrics, research impact assessment, and evaluation [10]. While librarians have existing professional knowledge that can be utilized, there are many concepts and tasks to master [11].

Thus, when implementing or augmenting research evaluation support services offered by the library, it is helpful to build a network of colleagues for support and discussion. Our group of seven libraries has come together to provide a network of support for each other. The group formed naturally based on frequent networking and conversations at conferences, webinars, and other events. We share new ideas and resources, we pose questions on metrics and measures, and we discuss issues with current tools and resources. Table 1 provides basic background information for each library. More detailed information about the background and future directions of services for each library can be found in the supplemental appendix to this paper.

**Table 1:** List of institutions and locations

Library/institution	Location	# of full-time professional library staff	# of full-time faculty or researchers	Institution type (private, public, government, funder)
Alberta Innovates-Health Solutions	Edmonton, AB, Canada	1	60*	Funder, Public
Galter Health Sciences Library & Learning Center, Northwestern University Feinberg School of Medicine	Chicago, IL, USA	20	2,059	Private
John W. Scott Health Sciences Library, University of Alberta	Edmonton, AB, Canada	7	1,060	Public
Samuel J. Wood Library, Weill Cornell Medicine	New York, NY, USA	18	1,762	Private
Lamar Soutter Library, University of Massachusetts Medical School	Worcester, MA, USA	13	1,348	Public
Becker Medical Library, Washington University in St. Louis	St. Louis, MO, USA	22	2,133	Private
US National Institutes of Health (NIH) Library	Bethesda, MD, USA	63	6,000	Government

* Research administration staff.

In response to increased communication and interest from other libraries, we created the Research Impact Services Google Group [12]. This group provides a collaboration and information forum for people who provide research impact assessment services. Group membership includes librarians, analysts, and visualization specialists.

## TYPE AND SCALE OF SERVICES

Libraries worldwide have chosen different models to implement research evaluation support services. Some libraries have formal services that exist as a department or specialized group in the library, while others have adopted a decentralized or informal approach where librarians provide services on an as-needed basis. At least one library suggests a pay-for-service model [13], while others have suggested providing a menu of options that outlines complimentary and fee-based services.

Everyday work in research evaluation services generally falls into two categories: reference support and consultation support. Reference support results in an email or conversation that summarizes any bibliometric findings in a very informal manner. Consultation support results in a formal product developed for the researcher or research group. Libraries with formal services provide reference and consultation services in a more programmatic way, either through training and education or by offering formal products as a service. Libraries with informal services can produce formal products but do not do so in a programmatic way.

For the group of seven libraries, we measured the scale of service based on the type of service provided and the number of formal products resulting from those services in one calendar year. A formal product is a document that highlights bibliometric activities of an individual, group, unit, center, or organization. It can include publication lists, traditional citation-based metrics, emerging article-level metrics, citation maps, coauthorship patterns, or other visualizations depicting bibliometric activity. A formal product includes a described methodology, year span, and sources used, with theoretically reproducible results. This document may be updated annually, but an annual update is not required for it to be considered a formal document.

Table 2 outlines the type and scale of evaluation services provided by the seven libraries, with the job titles and percentage time of those providing research evaluation support services. Taken together, the seven libraries showed diversity in the type and scale of services and in the types of jobs that support those services. These libraries created their research evaluation services to fulfill various needs, and in doing so, they each brought a unique context to the table. Of the four libraries that have formal services, the typical scale of services was robust. The three informal services all worked on a small scale. The job titles of those providing direct support for research impact–assessment activities varied among the libraries, with most libraries reporting two or more job titles. The percentage of staff time allocated to these activities coincided with the scale of services provided by the library; those with moderate or robust services allocated more staff time.

**Table 2:** Type and scale of services, including job titles and percentage of time

Library	Formal or informal services	Scale of services*	Job titles providing direct support	Percentage of time
Alberta Innovates-Health Solutions	Informal	Small	Embedded librarian	15%
Galter Health Sciences Library & Learning Center, Northwestern University Feinberg School of Medicine	Formal	Robust	Impact and evaluation librarian	100%
			Director of evaluation	20%
			Library director	10%
			Grant-supported project position	100%
John W. Scott Health Sciences Library, University of Alberta	Informal	Small	Public services librarian	20%
			Public services librarian	10%
			Reference collections assistant	5%
			Public services librarian	5%
Samuel J. Wood Library, Weill Cornell Medicine	Formal	Robust	Research impact and evaluation informationist	30%
			Scholarly publications librarian	80%
			Software developer	30%
			Identity services product manager	40%
			Library director	10%
Lamar Soutter Library, University of Massachusetts Medical School	Informal	Small	Head, Research and Scholarly Communications Services	20%
			Institutional repository librarian	10%
Becker Medical Library, Washington University in St. Louis	Formal	Moderate	Senior librarian	50%
			Scholarly publishing librarian	50%
NIH Library	Formal	Robust	Informationist	100%
			Informationist	100%

* Small scale consists primarily of reference or consultation services with 10 or fewer formal products per calendar year.

Moderate scale consists primarily of consultation services with 11–60 formal products per calendar year.

Robust scale consists primarily of consultation services with 60+ formal products per calendar year or the production of customized reports and tools for end users to create their own reports as desired.

As a complement to Table 2, descriptions of the scope of services at each library are provided below to illustrate the range of services available. These descriptions broadly capture core services and resources the libraries have created for their customer groups.

### Alberta Innovates-Health Solutions

The Alberta Innovates-Health Solutions (AIHS) Library supports approximately sixty research administration staff. Most of the library’s research impact services are provided for research grant programs managers and the Performance Management and Evaluation (PME) unit. A solo librarian serves the entire AIHS organization and is an active member of the PME unit. The librarian provides consultation, education, current awareness, and project support on research impact-related topics.

At the grant application adjudication stage, some peer-review committees and program managers request the compilation of research impact metrics as part of the input into the review process. For mid-grant or end-of-grant periods, the librarian verifies the research output reports (e.g., publications, patents, leveraged funding) as submitted by the grantees and occasionally gathers research impact metrics for aggregate reporting of programs. More complex bibliometrics projects are outsourced to external specialized bibliometrics consultants, with the librarian serving as a member of the project team or in an advisory role.

### Galter Health Sciences Library & Learning Center, Northwestern University Feinberg School of Medicine

Bibliometric and evaluation work at the Galter Health Sciences Library & Learning Center is coordinated through the library’s Metrics and Impact Core (MIC). The MIC provides services to faculty, staff, departments, institutes, and centers. Several resources have been developed to support evaluation work, including report templates for publication and citation data for individuals and groups as well as custom visualizations of that data upon request. The MIC has also created several guides and classes on topics such as tracking publications, enhancing research impact, and increasing visibility of research.

The MIC’s most requested services include providing guidance to centers and institutes on how best to track publication data of their members or trainees and to departments on how to better understand the impact of their research. The MIC works closely with the Evaluation and Continuous Improvement Program at the Northwestern University Clinical and Translational Science Institute (NUCATS), with campus leadership, and in collaboration with other groups in the scholarly environment such as the National Information Standards Organization, project collaborators, and vendor partners such as Digital Science.

### John W. Scott Health Sciences Library, University of Alberta

Until recently, research impact work was not something in which the John W. Scott Health Sciences Library was traditionally involved beyond answering reference questions about finding *h*-indexes or journal impact factors. In 2008, a contract librarian position was established and embedded in the Faculty of Nursing to meet the research needs of the faculty, including measuring whole-faculty research impact. The librarian established processes to routinely collect, track, analyze, and report faculty research impact. This position was also part of an evaluative group that explored the possibility of licensing current research information system products and other analytic tools available from Web of Science and Elsevier for the University of Alberta.

In 2015, the librarian’s contract position ended, and the librarian moved to the John W. Scott Health Sciences Library. The research impact work that was done by the librarian’s contract position is now performed by a team of four as a fee-for-service program. Since 2015, the librarian has continued supporting the information needs of the Faculty of Nursing and has performed additional research impact work for the Faculty of Medicine and School of Public Health through developing reports and leading workshops. The University of Alberta Library formed a Bibliometrics Working Group (from 2016 to 2017) that ultimately recommended that a full-time centralized bibliometrics librarian position be created for the University of Alberta. It is expected that this position will eventually lead a team of librarians to take on any research impact work currently being done by the library.

### Samuel J. Wood Library, Weill Cornell Medicine

The Samuel J. Wood Library and the C.V. Starr Biomedical Information Center hired a research impact and evaluation informationist in 2016 to provide answers to queries posed by researchers or administrators. These queries primarily seek ways to assess the impact of the extensive resources being poured into research in the past five years and how this research is reflected by Weill Cornell Medicine’s scholarly output and overall impact on the scientific knowledgebase. Increasingly common are questions focusing on the translation of scientific activities into clinical care.

Automated tools have become valuable in responding to these requests efficiently. Identifying the publications of faculty, students, and postdoctoral researchers is assisted by ReCiter [14, 15], a suite of automated tools developed at Weill Cornell for author name disambiguation. ReCiter leverages several types of institutionally maintained information about individuals to allow rapid and accurate assignment of publications to researcher profiles. To assess the scholarly impact of researchers at the institution, the program has been applying percentile rank–based metrics to times-cited data. Another tool developed at Weill Cornell, the citation impact tool (described later in this paper), is used to illustrate how the institution’s research enterprise has improved over the past five to ten years.

### Lamar Soutter Library, University of Massachusetts Medical School

The Lamar Soutter Library’s Research and Scholarly Communication Services Department provides informal research impact support that varies from providing productivity and impact reports upon request for faculty, departments, and administrators that can include a range of citation-based metrics, altmetrics, and/or collaboration data as appropriate; to presenting informational overviews of emerging metrics to various groups, and to developing online resources for the entire University of Massachusetts Medical School (UMMS) community. A publicly available library guide on research impact provides information, instruction, and links to resources for measuring impact. In addition, eScholarship@UMMS [16], the medical school’s institutional repository, provides monthly usage statistics to authors and research programs. For example, monthly dissemination statistics have been used by the University of Massachusetts Center for Clinical and Translational Science in their renewal application to demonstrate broader impacts of research.

### Becker Medical Library, Washington University in St. Louis

Becker Medical Library services related to research impact are available to all campus members. Becker Medical Library is seeing increased pressure for investigators to demonstrate the impact and value of their work—not just from external funding agencies, but also from university administrators as decisions are made relating to space allocation and tenure or promotion. Increasingly, investigators, funding agencies, and administrators are looking for information and data that will “tell a story.” The library provides coauthor network or geographic maps, which are especially helpful to illustrate impact and collaboration. Publication and citation reports (including various indexes such as *h*-index and *m*-index) and consultation on metrics are the most frequently requested services from Becker Medical Library.

Consultation also plays a large role in the scope of the library’s services. Becker Medical Library frequently provides guidance for administrators on appropriate metrics for benchmarking among academic groups to normalize for time, publication practices, types of faculty, and career length. Other consultation topics include the *h*-index, university or hospital ranking methodologies, and evaluation of trainees. Becker Medical Library also provides services to the Washington University Institute for Clinical and Translational Science as members of the Tracking and Evaluation Team.

### US National Institutes of Health (NIH) Library

The Bibliometric Services Program at the US National Institutes of Health (NIH) Library provides both standard and customized bibliometric services to NIH employees. Services provided through the program include consultations and advice on bibliometric approaches and methods, training on bibliometric theory and practice, standard bibliometric profiles of the intramural research produced by NIH institutes and centers, and customized bibliometric and portfolio analyses upon request by intramural and extramural staff. These customized analyses include bibliometric profiles of specific departments or grant portfolios, grant funding profiles for NIH institutes and centers, and landscape analyses to identify the major producers and research directions of publications in specific topics or disciplines.

## GOALS OF SERVICES AND TYPES OF PRODUCTS

There are many reasons a library may decide to provide research evaluation services. As a group, our libraries considered the types of goals that can be achieved by implementing these services. Each library reported how often those goals reflected the basis of their services (Table 3). The most frequently reported goals were related to providing bibliometric data to identify research impact or influence, answering reference questions related to research impact, and assisting faculty during the promotion and tenure process.

**Table 3:** Top reported goals of research evaluation services

Goals of research evaluation services	# of libraries reporting
Provide bibliometric data to help specific research groups, centers, or departments identify the impact of their research (e.g., citation data, coauthor citation mapping, etc.)	7
Provide the bibliometric impact or influence of specific publications	7
Provide the bibliometric impact or influence of specific efforts (e.g., grant or study)	7
Answer reference questions that deal with research impact	7
Provide assistance to faculty in the tenure and promotion process	5
Provide visualization services for research impact-related information	5
Provide educational services related to publication tracking and research discoverability as well as best use of citation databases	5
Advance the library’s mission through assisting researchers in decision making, promoting research results and impact, and furthering scholarly communications and bibliometric practice in the broader library community	5

To fulfill their goals efficiently and effectively, the libraries have developed reports, resources, and tools to support their services. The group of seven libraries has shared report templates, ideas for developing tools, and teaching materials with each other. Table 4 outlines the types of resources, guides, and tools that have been developed by the seven libraries. The libraries most frequently work on reports of publication activity, bibliometric reports for researchers or departments, and publication analysis of research areas. These resources, guides, and tools directly reflect the top goals reported by each library in Table 3, with bibliometric analyses making up the largest volume of work.

**Table 4:** Resources, guides, and tools developed

Resource, guides, and tools developed	# of libraries reporting
Mapping of publication activity (coauthor analysis)	7
Bibliometric report customized to needs of researcher or department	7
Publication analysis of a research area	6
Standard bibliometrics report	5
Annual organizational impact report	5
Website or web service	5
Online guide and tutorial (videos)	3
Teaching materials	3
Analysis of where organizational or discipline-specific researchers publish most to aid in collection development (for librarians)	2

## MARKETING OF SERVICES

Much thought is often given to strategic marketing of library services. Marketing can potentially take time away from doing work related to the service, but without marketing, key groups may not know a service exists. Therefore, it is imperative that any marketing plan have the most efficient approaches and target the appropriate groups. Table 5 indicates the top audiences for marketing services and the top marketing approaches across all potential groups.

**Table 5:** Top audiences and approaches for marketing services

Audience	# of libraries reporting	Marketing methods	#
Administration/institution*	7	Word-of-mouth	5
		Online resource	1
		Example products	1
Researchers	6	Word-of-mouth	5
		Orientation/presentation	1
Department chairs/departments	6	Example products	3
		Word-of-mouth	2
		Formal outreach	1
Program managers†	5	Word-of-mouth	3
		Example products	1
		Online resource	1
Research administration‡	5	Word-of-mouth	3
		Example products	1
		Orientation/presentation	1
Librarians	4	Word-of-mouth	2
		Example products	1
		Online resource	1
CTSA§ (US only)	4	Example products	2
		Formal outreach	2
Employees	3	Word-of-mouth	3
Students	2	Word-of-mouth	1
		Online resource	1

* Administration/institution is the top level of leadership in an organization, such as the president, provost, or dean of a particular school.

† Program managers coordinate, lead, and manage several related projects, often at the level of a university program or department, such as graduate programs in specific subject areas.

‡ Research administration collaborates with departments, centers, cores, and institutes to provide comprehensive services at essential steps of the research life cycle.

§ CTSA is the Clinical and Translational Science Award program by the US NIH, and universities receiving these awards often develop CTSA-related institutes to support research infrastructure and pilot funding (among other projects) on their campuses.

The seven libraries indicated that administrators, individual researchers, and departments were the groups most frequently identified as the audiences to whom research evaluation support services were marketed. Overall, word-of-mouth was a top marketing approach, with example products and online resources being the next most frequent approaches. Word-of-mouth might be a preferred method, because in general, research evaluation services were slightly more nontraditional than those typically found in libraries, and they might require an explanation that is tailored to the needs of specific audiences.

## COLLABORATIONS AND CUSTOMER GROUPS

**Figure 1:**
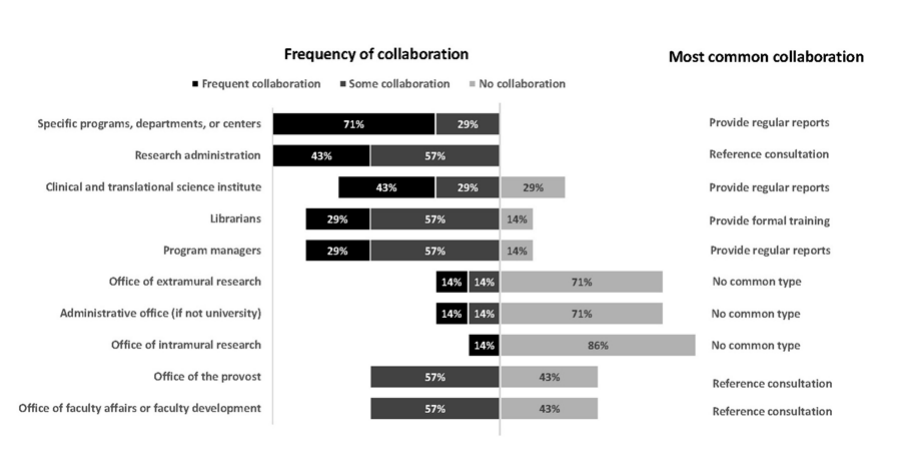
Type and frequency of collaborations with customer groups

Each library has evolved their services to reflect the needs of various customer groups. Figure 1 indicates the frequency of collaboration with specific groups and notes the most common type of collaboration or service. Examples of interesting projects that the libraries have completed with their collaborators are also provided to illustrate these collaborations. Collaborations and services include holding reference consultations to answer brief questions, providing formal training in one-off meetings, setting up regular meetings, providing reports, acting as coauthor on a final product, or being a named collaborator on a grant.

For the group of seven libraries, the most frequent interactions occurred with specific programs, departments, or centers; research administration; and clinical and translational science institutes. The most common collaboration type with any group was reference consultations to answer brief questions followed by providing regular reports and formal training.

Select evaluation support projects for each of the seven libraries are described below as a complement to Figure 1. The descriptions broadly discuss projects, customers, and final outputs.

### Alberta Innovates-Health Solutions

Research funding organizations face the challenge of tracking and gathering information about the research impact of their specific research funding. The organizations are not able to rely on acknowledgment information in publications, as this information is not consistently reported or presented in the metadata of publications.

AIHS often relies on reporting of publications or other scholarly outputs directly from the grantees, though inevitably underreporting remains an issue. A program supported by AIHS and their collaborating funder recently requested assistance in learning about the impact of their program. The librarian worked with a staff evaluator and gathered bibliometric data from Scopus on all of the program’s grantees’ reported articles (e.g., citations, coauthors). Through this work, the librarian was able to identify highly cited, top, and hot papers by the program’s grantees using the InCites Essential Science Indicators database. The staff evaluator performed citation analysis, geographical mapping of the citing authors from various countries, and an analysis of other reported outcomes (e.g., number of research trainees supported by the program). The bibliometric analysis enhanced the report showcasing the impact of this funding opportunity.

### Galter Health Sciences Library & Learning Center, Northwestern University Feinberg School of Medicine

In 2014, the MIC supported NUCATS with their grant renewal process. Four librarians helped with the work: the library director (who also serves as the director of evaluation for NUCATS), the biosciences and bioinformatics librarian, a research librarian, and the impact and evaluation librarian. In cooperation with NUCATS, the MIC developed a strategy to identify and link publications that should have been associated with the CTSA as part of the publishing workflow. The librarians first searched NIH RePORTER for the number of linked publications for each CTSA hub so that they would have a better sense of the potential scope of the missed links to publications in comparison with each hub. The librarians provided guidance in tracking publications related to the grant and direct support in linking appropriate publications to the grant using NCBI’s MyBibliography.

The librarians used the metadata associated with those publications to create visualizations of their research impact, provide evidence of NUCATS collaborations with other CTSAs, and showcase their intense productivity during the past award period. The MIC worked alongside NUCATS leadership over the five months that led up to the renewal deadline, and the strong partnership continues today in providing educational and training services for NUCATS trainees, ongoing publication tracking services, and customized reports on publication data related to NUCATS.

### John W. Scott Health Sciences Library, University of Alberta

The John W. Scott Health Sciences Library wanted to better understand the level of expertise and amount of effort required to support researchers in competing for research funding. To learn more, the librarian supporting the Faculty of Nursing approached the university’s grant assist office with a proposal to work on a bibliometrics project for a researcher who was likely to be applying for funding in the near future. The librarian was matched with a potential grantee who was applying for funding from the Canadian Institutes of Health Research (Canada’s national health research funder).

The researcher was very enthusiastic about receiving library support in this area. The librarian created a research-impact profile document that reported on a range of bibliometric research impact analyses using various literature databases. The profile document had both visual and textual data, including coauthorship data, citation data, and collaboration data. The overall project was relatively time-intensive, and completing the research impact profile document took the librarian five working days. The profile document was well received by the researcher, and the librarian was asked by the grant assist office to conduct ten to fifteen more research impact profiles.

Ultimately, the library concluded that they did not have enough capacity to provide this level of support to researchers. Currently, there is not a specific position dedicated to research-impact work in the whole library system or at the John W. Scott library. However, the University of Alberta Libraries are planning to develop a centralized bibliometrics team to take on this type of work. Overall, the project affirmed that there is a strong demand for research impact work from the library.

### Samuel J. Wood Library, Weill Cornell Medicine

In 2014, the Weill Cornell Medicine Graduate School and Research dean began requesting reports on the citation impact of specific researchers. Given the significant time required to create these reports manually, a group was convened to develop an automated system. The product of these efforts was the citation impact tool, a system for calculating and visualizing citation impact data. The system calculates any scholarly article’s percentile rank of times cited measured against a baseline of articles of the same type, in the same field, and published the same year. The system presents this information visually as an iconographic box plot, portraying a researcher or department’s profile of articles as a collection, with each article displayed in a bin corresponding to its normalized percentile rank.

The team consisted of the library director; an informationist in the research services unit; an identity services product manager; a scholarly librarian from the Information, Education, and Clinical Services Unit; and two developers from the Application Development and Analytics Unit. The code [17] has been released publicly, and the team hopes to work with institutional partners to implement the tool, which might eventually allow for cross-institutional comparisons of citation impact.

### Lamar Soutter Library, University of Massachusetts Medical School

In 2014, shortly after acquiring Scopus and SciVal, the library’s Research and Scholarly Communication Services Department reached out to different departments about research impact, including the Department of Emergency Medicine. Upon seeing the potential of these tools for benchmarking, the department’s director of research—who, at the time, was looking for a method to evaluate the department’s research performance against that of other emergency medicine departments—initiated a large-scale, collaborative benchmarking project with the library. The project was designed to evaluate and rank the department and its faculty against its peers at other institutions on the basis of productivity (output over time) and impact (citations, cited publications, and citations per publication) measures.

Using data from Scopus and SciVal, the project team generated three-year metrics for ten randomly selected faculty from ten randomly selected peer institutions and UMMS. The results showed both strengths and areas for improvement for the group and, more importantly, stood out as a model process for research performance evaluation. The Department of Emergency Medicine has since expanded this project to evaluate the department compared to all emergency medicine departments in the United States. One librarian worked together with the emergency medicine director of research and a research assistant over the course of six months to complete the primary benchmarking phase of this project.

### Becker Medical Library, Washington University in St. Louis

One example of a rewarding project for the Becker Medical Library was a faculty request to provide visual evidence of collaboration among a research group over a ten-year period to demonstrate coalescence over time. Scopus was used to collect the publication data, and the Science of Science (Sci2) tool (Indiana University and SciTech Strategies) [18] was used to create a coauthor network to visualize collaboration patterns over a ten-year period. The network image was used in a successful funding renewal application and led to increased awareness of our services on campus, including three referrals.

### NIH Library

One of the most interesting projects worked on by the Bibliometrics Services Program at the NIH Library was to map the research topics of NIH intramural research. Using data from Web of Science, they combined citation- and text-based methods using the Sci2 tool and Gephi to identify the research topics of papers published by intramural researchers at fifteen NIH institutes from 2008 to 2012. For each institute, they created a bibliographic coupling network, in which papers were connected if they shared references with each other, and then used a network-based community-detection algorithm to identify clusters of papers that shared references more frequently with each other than they did with other papers in the network.

They then performed word co-occurrence analysis on the titles of the papers assigned to each cluster to identify the topics of the papers in each cluster. The results identified both the unique strengths of each institute and potential areas of topical overlap among institutes. These results could also be used to facilitate communication and collaboration across the NIH intramural research community to either identify potential research collaborators at other institutes or to reduce overlaps in the research performed at different institutes.

## COMMONLY USED TOOLS AND RESOURCES

**Figure 2:**
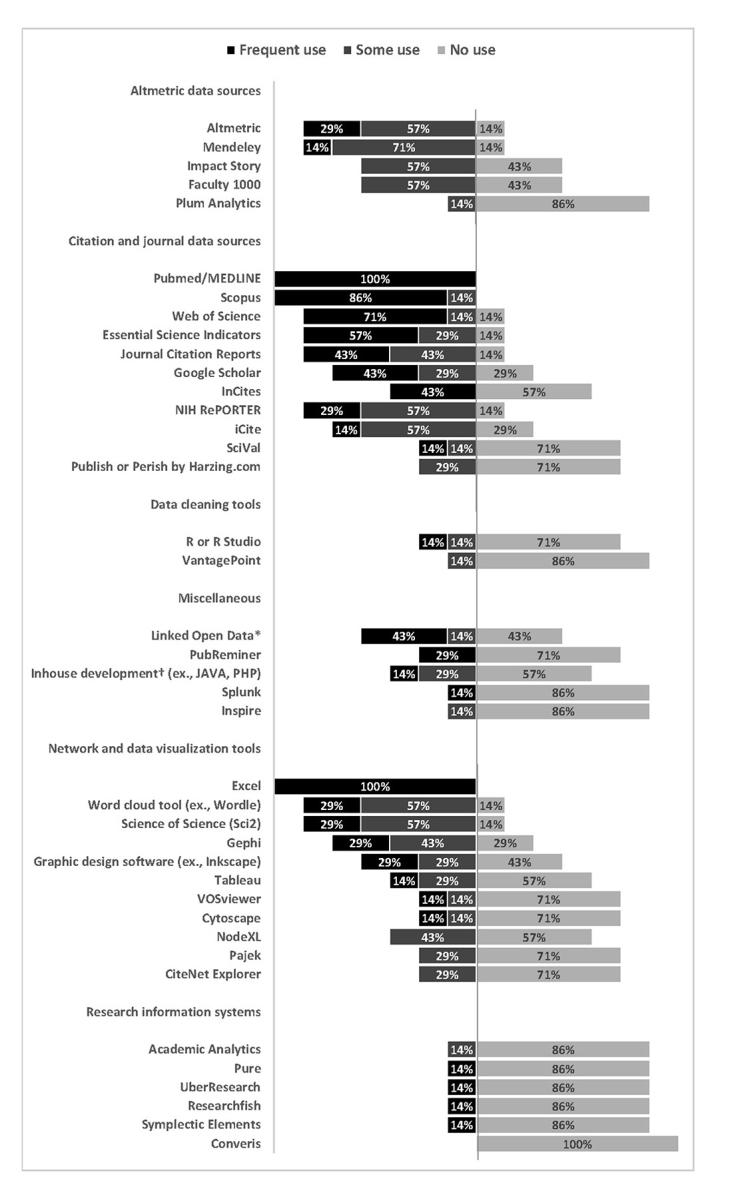
Tools, systems, and data sources used to provide services and frequency of use * Linked open data is a way of publishing structured data that allows metadata to be connected and enriched so that different representations of the same content can be found and links made between related resources [19]. ^†^ In-house development refers to a product created internally rather than obtained from a third party. Examples include writing PHP or Javascript to gather, clean, or analyze data.

There are many commonly used tools for providing research evaluation services. However, the cost of some tools makes them unreachable for some libraries. Our seven libraries have access to various proprietary and freely available tools. We report on the frequency of use of tools, systems, and data sources used to provide services in Figure 2. The most intensely used tools (those that at least four libraries have indicated that they always use) are PubMed/MEDLINE, Scopus, and Web of Science.

## CONCLUSIONS

Librarians at a wide range of medical libraries are increasingly being asked for assistance with assessing the value and impact of scholarly research. The libraries surveyed here have responded to this demand in different ways, depending on their local contexts. Although there are similarities in the data sources (e.g., PubMed, Scopus, Web of Science) and tools (e.g., Excel, Sci2, Gephi) used by each library, the audiences and applications of those data sources and tools vary among libraries depending on the specific needs of their respective institutions. This suggests that although there may be a common bibliometric skill set that librarians who are involved with bibliometric analyses share, the actual applications of those skills are driven by the needs of their institutions and may, therefore, appear quite different at various institutions.

The survey also identified a trend in the amount of staff time available to perform bibliometric analyses. Libraries with less robust programs tend to focus on ad hoc individual- and laboratory-scale impact and benchmarking projects, whereas libraries with more robust programs tend to focus on institution-scale analyses and systems. Libraries with less formal programs also highlight the need to add capacity in the form of additional staff time or new positions to expand their services. This suggests that the complexity and scale of bibliometric analyses requested from and performed by librarians is directly proportional to the amount of staff time available to perform them. This also suggests that the more a library invests staff time in bibliometric analyses, the more its institution tends to ask of it.

Finally, the success of the programs highlighted here suggests that conducting bibliometric analyses is a service opportunity for medical librarianship as a profession. While many medical librarians have been providing ad hoc research impact services for years, the rapid growth of library programs in this survey suggests the value in formalizing and advancing these services in medical librarianship more generally. The success of these programs demonstrates that there is a demand for bibliometric services at medical institutions and that these institutions are willing to turn to libraries to meet that demand. We recommend that librarians, libraries, and library associations embrace bibliometric services in the same way that they have embraced data management services as a way to advance the profession of librarianship and provide greater value to our institutions.

Adding bibliometric analyses to the suite of services provided by librarians will take concentrated effort. It will require a substantial training initiative to ensure librarians have the skills and knowledge to perform bibliometric analyses that are both accurate and appropriate. Some libraries in this survey have begun offering or organizing training in bibliometric analyses for librarians, but more is needed. It will also require additional knowledge sharing and collaboration among libraries to establish practice guidelines for providing bibliometric services. The librarians included in this survey have met mostly by chance and have communicated either directly with each other or through the informal Research Impact Google Group [12] set up for this purpose, but a more formal special interest group would be beneficial to facilitate discussion around bibliometric analyses in libraries. The scope of the opportunities inherent in bibliometric analyses and the success of the programs highlighted in this survey suggest that such change is worthwhile for libraries, the profession of librarianship, and, most importantly, our customers.

## Supplemental File

Appendix Background information and future directions
