# Supplementary material for: Research evaluation support services in biomedical libraries
Source: J Med Libr Assoc. 2018 Jan 2;106(1):1–14. doi: 10.5195/jmla.2018.205 (PMC5764574; doi:10.5195/jmla.2018.205)
Supplement: Appendix [file jmla-106-1-s001.pdf]

JMLA Journal of the Medical Library Association

SC
Supplemental content to
J Med Libr Assoc. Jan;106(1):dx.doi.org/10.5195/jmla.2018.205
www.jmla.mlanet.org
© Gutzman, Bales, Belter, Chambers, Chan, Holmes, Lu, Palmer, Reznik-Zellen, Sarli, Suiter, Wheeler 2018

Research evaluation support services in biomedical libraries

Karen Elizabeth Gutzman; Michael E. Bales; Christopher W. Belter; Thane Chambers; Liza Chan; Kristi L. Holmes; Ya-Ling Lu; Lisa A. Palmer, AHIP; Rebecca C. Reznik-Zellen; Cathy C. Sarli, AHIP; Amy M. Suiter; Terrie R. Wheeler

APPENDIX

Background information and future directions

Each of the seven libraries provides basic background information about their customer groups and the formation of their services. Each library has provided some ideas for the future directions of their services. These ideas generally fall into two categories: those that are concerned with institutional integration and those that are concerned with tool and policy development. Institutions with smaller informal services are more concerned with institutional integration; those with larger, more formal services are focused on tools and policies.

Alberta Innovates-Health Solutions

Alberta Innovates-Health Solutions (AIHS) Library is a Canadian-based, publicly funded, not-for-profit, provincial organization mandated to improve health, the health system, and socioeconomic well-being of Albertans through health research and innovation. AIHS has fervent interest and involvement in research impact assessment, from testing the "Payback Model" in its former organization [1] to developing and implementing a research impact framework [2] based on the Canadian Academy of Health Sciences framework [3], and more recently, hosting the International School on Research Impact Assessment and the local iterations of the course. The AIHS library is staffed by a solo librarian, and she supports the various research impact assessment activities throughout the research investment life cycle.

As of November 1, 2016, AIHS merged with the other three Alberta Innovates corporations (which focus on agriculture, energy, forest, and environment sectors) to become one consolidated Alberta Innovates agency that spans research, innovation, and commercialization support in areas beyond health. With the expanded subject coverage, it is expected that the research impact assessment activities will require more intense library support for impact metrics that point toward policy, economic, and societal changes.

Galter Health Sciences Library & Learning Center, Northwestern University Feinberg School of Medicine

The Galter Health Sciences Library & Learning Center is a fully integrated information and knowledge management hub for the Northwestern University Feinberg School of Medicine. In 2014, the library launched the Metrics and Impact Core (MIC) to formalize a comprehensive suite of services related to research evaluation and impact assessment, benefitting the entire enterprise. The Galter Library is uniquely positioned as a center in the Northwestern University Clinical and Translational Sciences Institute (NUCATS), allowing important synergies between the research, clinical, and education activities of Galter Library and NUCATS. This strong partnership has enabled the MIC and other library programs to receive adequate support and attention across the departments, centers, researchers, and faculty at the university. The Galter Library has also implemented an institutional repository with machine-readable metadata, DigitalHub [4], to facilitate the discovery and recognition of research and scholarship at Northwestern Medicine. Through their work with NUCATS and in coordination with campus leadership, they have supported innovation and acceleration of scientific discovery across the enterprise.

The MIC collaborates with centers and institutes to design evaluation programs, build evaluation effort into their funding models, and grow a culture of evaluation and continuous improvement on campus. Through the work with NUCATS and, later, with the Chicago Cancer Health Equity Collaborative (ChicagoCHEC), the MIC has standardized evaluation and reporting processes and applied

SC
Supplemental content to
J Med Libr Assoc. Jan;106(1):dx.doi.org/10.5195/jmla.2018.205
www.jmla.mlanet.org
© Gutzman, Bales, Belter, Chambers, Chan, Holmes, Lu, Palmer, Reznik-Zellen, Sarli, Suiter, Wheeler 2018

results-based accountability principles [5] broadly to help groups identify metrics that matter; several efforts are underway that leverage these workflows. The MIC is growing their web presence and beginning to provide just-in-time resources for the groups they serve. Ultimately, the MIC aims to build a campus-wide evaluation infrastructure and hope to bolster their data and statistical methodologies and grow their evaluation specialty services to include expertise in advanced data visualization, economic analysis, and social and policy impact.

John W. Scott Health Sciences Library, University of Alberta

The John W. Scott Health Sciences Library is part of the University of Alberta Library system, which comprises 9 subject-focused libraries. The library serves over 8,000 pre-licensure, graduate students in health sciences programs spanning 9 faculties, plus research and teaching staff, and over 900 postgraduate residents and fellows. The 5 main health sciences faculties are: Medicine and Dentistry, Nursing, Pharmacy and Pharmaceutical Sciences, School of Public Health, and Rehabilitation Medicine.

Currently, two librarians are part of a Research Impact Working Group that will develop recommendations in early 2017 for the senior library administrative team on if and how they can move forward in this area. The continuation of intense work in the area of research assessment will lie in decisions made from these recommendations.

Samuel J. Wood Library, Weill Cornell Medicine

Weill Cornell Medicine is among the fastest-growing medical school and research universities, doubling its physical research footprint in 2014 [6]. To best serve the institution's rapidly growing research, clinical care, and medical school missions, the Samuel J. Wood Library and C.V. Starr Biomedical Information Center began offering informal bibliometrics services in 2009, when they were first funded by the National Institutes of Health (NIH) VIVO grant. The foundation for a formal service was established in 2014, and a research impact and evaluation informationist was hired in 2016. Anticipating that the establishment of a formal service would generate additional service requests from administrators and investigators and that the number of such requests would far exceed what could be managed manually, the service has been developing automated tools through the VIVO program.

The library has openly shared the code for both ReCiter [7, 8] and VIVO Dashboard [9], so that other organizations can use similar methodologies to accomplish this work. They further plan to continue development of automated approaches to compile and analyze data for research impact evaluation.

Lamar Soutter Library, University of Massachusetts Medical School

The University of Massachusetts Medical School is the state's first and only public academic health sciences center and includes NIH-funded UMass Center for Clinical and Translational Science. The Lamar Soutter Library serves over 3,000 faculty (full-time, part-time, and volunteer) and over 1,000 students in the Schools of Medicine, Nursing, and Biomedical Sciences, and the clinical system. The library established the Research and Scholarly Communication Services Department in 2009, after a strategic planning process, to provide outreach and support for issues related to scholarly publishing and open access. The department incorporated the institutional repository (eScholarship@UMMS) [10], which was established in 2006, as well as the ongoing work that had been done by reference librarians providing *h*-index reports for various faculty and administrators. In 2013, the Research and Scholarly Communication Services Department began outreach on altmetrics and research impact, and in 2014, the library acquired Scopus and SciVal, enabling more detailed support for research evaluation. Throughout this time, the department has continued to provide ad hoc bibliometric reports for individuals and departments.

Moving forward, the primary goal for Research and Scholarly Communication Services is to formalize the otherwise ad hoc efforts at research impact support into a programmatic and formalized service. This would include standardizing reports for faculty and administrators, acquiring needed

SC
Supplemental content to
J Med Libr Assoc. Jan;106(1):dx.doi.org/10.5195/jmla.2018.205
www.jmla.mlanet.org
© Gutzman, Bales, Belter, Chambers, Chan, Holmes, Lu, Palmer, Reznik-Zellen, Sarli, Suiter, Wheeler 2018

impact tools and additional staff, and informing the faculty evaluation processes at the medical school. The department also looks forward to continuing their partnerships with other offices on campus to promote and encourage ORCID adoption, which is an important and growing component of research impact.

Becker Medical Library, Washington University in St. Louis

The Becker Medical Library serves a school of medicine campus comprising over fifty academic departments, programs, divisions, specialized research centers, hospitals, and the Institute for Clinical and Translational Science (ICTS), funded by the National Center for Advancing Translational Sciences. The Becker Medical Library Publishing and Evaluation program provides services and resources for all campus members. The program traces its genesis to a simple question as to number of citations for publications generated by a research study. This question led to a retrospective analysis of the Ocular Hypertension Treatment Study (OHTS), which was completed in 2007 using bibliometric-based methods. The project further expanded to identify and locate evidence of impact beyond use of publication metrics as well as review of publication practices by OHTS investigators. The findings raised questions regarding the suitability of bibliometric-based methods to illustrate diffusion of research into meaningful outcomes, such as synthesis into clinical practice and influence on public policy, among others. This project provided the impetus for the Becker Medical Library to offer a program to supplement traditional services to provide a more contextual narrative of impact. Establishing a publishing and evaluation program was a compelling and strategic direction for the library as it provided staff the opportunity to align their skills and expertise with the immediate needs of the campus.

New directions for the Becker Medical Library include identifying appropriate bibliometric-based analyses for specific purposes, learning new software applications, and developing standard procedures for program activities and reports.

US National Institutes of Health (NIH) Library

The US National Institutes of Health (NIH) Library is one of the largest biomedical research libraries in the United States. Its collections and services are developed to support the programs of the NIH and selected US Department of Health & Human Services agencies. The library provides access to over 10,000 electronic journals, 16,000 e-books, and 170 databases. The NIH Library's service areas include bibliometrics, bioinformatics, custom information solutions, data management and analysis, 3-D printing, document delivery, editing, literature searching, informationists, systematic reviews, training, and translations.

In the future, the NIH Library plans to create a policy for the responsible use of bibliometrics in research assessment and decision making to provide recommendations on how analyses should be understood and used. Other plans include providing additional training opportunities for librarians who are interested in bibliometrics, expanding the range of services provided by the library, and pursuing the use of technology, especially code, to increase efficiency, enable scale, and add capacity to NIH Library services.

References

1. Buxton M, Schneider W. Assessing the "payback" from AHFMR-funded research. Edmonton, AB, Canada: Alberta Heritage Foundation for Medical Research; 1999.
2. Graham KER, Chorzempa HL, Valentine PA, Magnan J. Evaluating health research impact: development and implementation of the Alberta Innovates-Health Solutions impact framework. Res Eval. 2012;21:354–67.
3. Canadian Academy of Health Sciences. Making an impact: a preferred framework and indicators to measure returns on investment in health research. Ottawa, ON, Canada: The Academy; 2009.

SC
Supplemental content to
J Med Libr Assoc. Jan;106(1):dx.doi.org/10.5195/jmla.2018.205
www.jmla.mlanet.org
© Gutzman, Bales, Belter, Chambers, Chan, Holmes, Lu, Palmer, Reznik-Zellen, Sarli, Suiter, Wheeler 2018

4. Galter Health Sciences Library & Learning Center. DigitalHub [Internet]. Chicago, IL: The Library [cited 19 Oct 2017]. <https://digitalhub.northwestern.edu/>.
5. Friedman M. Trying hard is not good enough: how to produce measurable improvements for customers and communities. Victoria, BC, Canada: Trafford Press; 2005.
6. Sunkin-Strube A. Weill Cornell Medicine thrives in decade of growth. EZRA Cornell Universitys Magazine [Internet]. Spring 2016 [cited 19 Oct 2017]. <http://ezramagazine.cornell.edu/SPRING16/CoverStorySidebar6.html>.
7. Albert P, Bales M. ReCiter: GitHub [Internet]. 2014 [cited 17 Oct 2017]. <https://github.com/wcmc-its/ReCiter>.
8. Johnson SB, Bales ME, Dine D, Bakken S, Albert PJ, Weng C. Automatic generation of investigator bibliographies for institutional research networking systems. J Biomed Inform. 2014 Oct;51:8–14.
9. Weill Cornell Medicine. Citation impact tool: GitHub [Internet]. Weill Cornell Medicine [cited 17 Oct 2017]. <https://github.com/wcmc-its/vivodashboard>.
10. Lamar Soutter Library. eScholarship@UMMS [Internet]. Worcester, MA: University of Massachusetts Medical School [cited 17 Oct 2017]. <http://escholarship.umassmed.edu>.
